# The Role of Flexibility in the Formation and Maintenance of Extreme Beliefs: A Narrative Scoping Review

**DOI:** 10.1111/sjop.70110

**Published:** 2026-05-02

**Authors:** Sebastian Deverson, Paul Delfabbro, Ryan Balzan

**Affiliations:** ^1^ School of Psychology University of Adelaide Adelaide South Australia Australia; ^2^ College of Education, Psychology and Social Work Flinders University Adelaide South Australia Australia

**Keywords:** affective flexibility, belief flexibility, cognitive flexibility, extreme overvalued beliefs, extremism, psychological flexibility

## Abstract

There is growing interest in the role of cognitive flexibility in the development of politically motivated violence and a willingness to self‐sacrifice. However, the construct of flexibility has been applied in multiple ways across disciplines, resulting in conceptual ambiguity and methodological challenges. This narrative scoping review re‐evaluated the theoretical mechanisms linking distinct flexibility constructs to extreme beliefs, with the aim of clarifying their conceptual boundaries to direct future research. A narrative synthesis was conducted to identify and integrate theoretical accounts of flexibility relevant to extreme belief formation and maintenance. Four flexibility variants were identified as relevant: cognitive, affective, psychological, and belief flexibility. Each was conceptualized as facilitating updating and switching within cold, decontextualized cognitive tasks or hot, contextualized affective tasks to enable goal‐directed problem solving. These abilities were not found to promote a single problem‐solving strategy. Rather, they enabled a range of adaptive responses, including evidence integration, critical thinking, emotion regulation, and tolerance of uncertainty. Their use was associated with effective navigation of conflicting evidence, openness to new ideas and perspectives, and reflective awareness of emotional responses and cognitive biases. The findings indicate that flexibility operates across both affectively charged and emotionally neutral cognitive processes, the activation of which depends on the emotional valence of the context. In this way, cognitive and affective flexibility appear to function as complementary protective factors against the development of extreme beliefs. Future research should examine how these forms of flexibility interact across time in belief formation and maintenance.

## Keypoints

- Four flexibility variants were identified as relevant to extreme beliefs, including cognitive, affective, psychological, and belief. Each contributed uniquely to belief formation and maintenance processes.
- Flexibility was identified as a higher‐order ability that enables adaptive cognitive strategies within complex, politically and ideologically charged contexts.
- Cognitive and affective flexibility appeared to function as complementary protective factors against extreme beliefs, with their activation dependent on the emotional intensity and contextual features of the environment.

## 1 Introduction

### 1.1 Rigidity of the Right

A meta‐analysis comparing liberals and conservatives along multiple aspects of cognition suggests clear differences between the moderate political left and right (Jost 2017; Jost et al. 2017). Indeed, among the 133,796 participants from 14 countries, conservatives demonstrated significantly greater dogmatism, need for certainty and closure, intolerance of uncertainty, and cognitive rigidity compared to liberals. Liberals further exhibited a greater need for effortful reasoning and cognitive reflection. It is argued, however, that these differences diminish at the extreme ends of the political spectrum, where dogmatism and intolerance become particularly salient irrespective of the side of politics (Brandt et al. 2014; van Prooijen and Krouwel 2017). Recently, cognitive rigidity or inflexibility, or the inability to adapt one's thoughts, strategies, and behaviors to enable effective goal‐directed problem‐solving, has been considered to influence extreme partisanship across the political spectrum (Zmigrod and Rentfrow 2020). Moreover, cognitive inflexibility is associated with the endorsement of violent extremism, including a willingness to fight and self‐sacrifice on behalf of group members and shared values (Schumann et al. 2022; Zmigrod 2022; Zmigrod, Rentfrow, and Robbins 2019).

Notwithstanding these emerging findings, there has generally been limited investigation into the role of rigidity in the development and maintenance of the strongest of group ideological beliefs, or extreme beliefs that endorse the use of violence and sacrifice to achieve an ideological, religious or political goal (Haq et al. 2020; McCauley and Moskalenko 2008). Most commonly, rigidity has tended to be investigated in the context of prejudice, ethnocentrism and right‐wing authoritarianism. Such work suggested that right‐wing authoritarianism was the result of rigid cognition, or an overreliance on previously used, embedded or “mechanized” cognitive strategies that fail to be updated (Adorno 1950; Rokeach 1948, 1951). Those who applied rigid cognition were persistent in their problem‐solving strategy and engaged in black‐and‐white thinking (Rokeach 1951; Van Hiel et al. 2010). Recent research on executive functions and cognitive flexibility supports these early findings, in that reduced executive functioning is negatively associated with openness to changing fundamentalist religious beliefs and positively with a variety of conspiracy theories (Muluk et al. 2020; Sambol et al. 2024).

### 1.2 Flexibility and Extreme Beliefs

Despite the long history of research, there is no concise model or theory that relates rigidity and flexibility to extreme belief formation and maintenance. There are, however, several theoretical mechanisms that describe how flexibility can be applied to protect against extreme beliefs. For example, flexibility enables greater perception of changes in context to a problem, perception of new information, and the ability to restructure existing knowledge and strategies (Diamond 2013; Spiro and Jehng 1990). Flexibility additionally enables comprehension of the abstract elements of a scenario (i.e., patterns, themes or objectives), as well as an ability to perceive similarities in abstractions across thematically related contexts (Briell et al. 2011). The relevant knowledge can then be creatively integrated into a cognitive strategy to match the demands of a given scenario and achieve the intended outcome. Contrastingly, rigid individuals lack the ability to notice nuance and changes in their environment; and fail to employ alternative strategies when the context changes and an intended solution is not achieved (Channon 1996). Rigidity further limits creative integration by encouraging the use of embedded strategies, knowledge and perspectives (Baron 2019; Mednick 1962).

Furthermore, flexible people are aware of the problem‐solving strategies they apply to direct cognition, while remaining critical and open to applying a wide variety of strategies (Garcia‐Mieres et al. 2018; Pennequin 2022). Beliefs can be considered flexible, therefore, when they are held with a degree of awareness, are assessed critically, and can be updated where necessary. Contrastingly, rigid beliefs may be those that rely on singular, categorical perspectives and methods of information processing, that also fail to be updated. These can inevitably begin to reflect an outdated or inaccurate assessment of reality (Rollwage et al. 2018). An additional function of flexibility is the ability to admit the possibility that a cognitive strategy, perspective or belief is wrong (Diamond 2013). It is thus unsurprising to find that as rigidity increases when exposed to threat, intergroup biases and conviction in a belief's validity similarly increase (McGregor et al. 2001). Greater flexibility is further associated with other cognitive functions known to be protective of extreme beliefs. Openness to experience, for example, is negatively associated with preference for right‐wing authoritarianism (Jost et al. 2003; Riemann et al. 1993). For these reasons, we may consider flexibility to be a potential underlying protective factor against the development of extreme beliefs.

### 1.3 Flexibility Applications

However, these theoretical mechanisms have their limitations. Complications arise when considering the various applications of flexibility across different domains. Cognitive flexibility, a core executive function, refers to the ability to update and switch between one's thoughts, strategies and behaviors to enable effective problem‐solving and goal‐directed behavior within decontextualized, emotionally neutral tasks (Dajani and Uddin 2015; Diamond 2013). However, other applications of flexibility (e.g., belief flexibility and psychological flexibility) extend beyond this definition, and are occasionally conflated with similar constructs (e.g., inhibition and self‐regulation). Additionally, the various applications describe unique mechanisms that may contribute to extreme belief development, which make it difficult to infer a core or foundational construct upon which to base future research. For example, affective flexibility describes difficulties in switching between emotionally charged and neutral stimuli (Genet et al. 2013; Kraft et al. 2020; Twivy et al. 2021). Psychological flexibility, a central tenet in Acceptance and Commitment Therapy (ACT), shares similarities with cognitive flexibility (Cherry et al. 2021), but also emphasizes tolerance and acceptance of distressing emotions and experiences (Burton and Bonanno 2016; Kashdan and Rottenberg 2010). In addition, belief flexibility involves to the ability to integrate novel or disconfirmatory evidence to update a belief (Woodward et al. 2006). Although belief flexibility has been investigated in relation to delusions and schizophrenia, evidence suggests that non‐clinical populations may demonstrate belief rigidity under some conditions as well (Cesur et al. 2023; Colbert et al. 2010; Zhu et al. 2021).

### 1.4 The Present Study

At present, the theoretical mechanisms relating flexibility and rigidity to extreme beliefs fail to account for several important factors. For instance, researchers have relied on prejudice research to draw connections between flexibility and extreme beliefs. This is problematic given that extreme beliefs and behaviors do not necessarily develop linearly from prejudice (McCauley and Moskalenko 2017; Pilkington 2023). Furthermore, it is unclear how various applications of flexibility, especially those applied to emotional processes, relate to extreme beliefs. It is also unclear if rigidity at the extremes is indicative of a generic rigidity across applications, or if a person can be rigid in one application and flexible in another. These gaps in our knowledge present a challenge in understanding how extreme beliefs are developed and maintained, and it is therefore important to re‐evaluate the role of flexibility and rigidity in this context. For these reasons, the broad goal of this narrative scoping review was to provide an overview of the empirical and theoretical evidence connecting various applications of flexibility to extreme belief formation and maintenance. Accordingly, the review explored several flexibility applications known to relate to beliefs in some capacity, including cognitive flexibility, affective flexibility, belief flexibility, and psychological flexibility.

It should be noted that the review was designed to cover a broad range of flexibilities to direct future research in the area. Consequently, each flexibility was briefly discussed and only covered the main findings. We accounted for this broad study design in several ways. First, only applications of flexibility that have been related to beliefs and politically motivated behavior were discussed. Second, this review deliberately excluded similar beliefs that are theoretically connected but fundamentally distinct (i.e., clinical constructs such as delusions and obsessions).

## 2 Method

### 2.1 Inclusion Criteria

Study selection was guided by whether flexibility was measured or meaningfully discussed in relation to extreme overvalued beliefs or closely related constructs, including authoritarianism, political violence, and dogmatic or intolerant attitudes. Studies examining flexibility within clinical or subclinical populations, such as those experiencing psychotic‐like symptoms or delusional beliefs, were included only where a non‐clinical control group was present, which allows comparison with populations more reflective of the focus of this review.

Inclusion criteria were intentionally broad given the emerging and conceptually diverse nature of the field. Studies were excluded if flexibility was not operationalized as a central variable, or if it was only mentioned briefly without measurement or substantive discussion. Gray literature was excluded. No restrictions were placed on political orientation, ideology, or demographic characteristics such as age, gender, education, or geographic location. Similarly, no limits were set on study design or measurement type, as the review aimed to examine how flexibility is conceptualized and assessed across methodological approaches.

### 2.2 Screening Procedure

The scientific literature was systematically searched for studies examining flexibility in relation to extreme overvalued beliefs and related constructs. Searches incorporated both specific flexibility constructs and broader terminology. Specific constructs included cognitive flexibility, affective flexibility, psychological flexibility, explanatory flexibility, and belief flexibility. These categories were selected because they represent distinct theoretical operationalizations of flexibility that have been examined in research on extreme beliefs, delusions, or related factors such as distress, openness to experience, resilience, and critical thinking. Flexibility was also searched using the broader term flexibility to capture studies that did not adopt subtype labels.

Searches were conducted in Ovid including EMBASE and PsycINFO, as well as PubMed and Scopus. Title, abstract, and keyword fields were searched where available. Search terms relating to flexibility were combined with terms indexing extreme beliefs and related constructs, including authoritarianism, overvalued ideas, overvalued beliefs, irrational beliefs, extreme beliefs, delusions, conspiracy theories, ideology, and ideological beliefs. Truncation symbols were used where appropriate to capture variations of inflexibility and related terms. Search parameters were adapted to the requirements of each database and were limited to social sciences, psychology, and health professions where possible.

### 2.3 PRISMA

**FIGURE 1:**
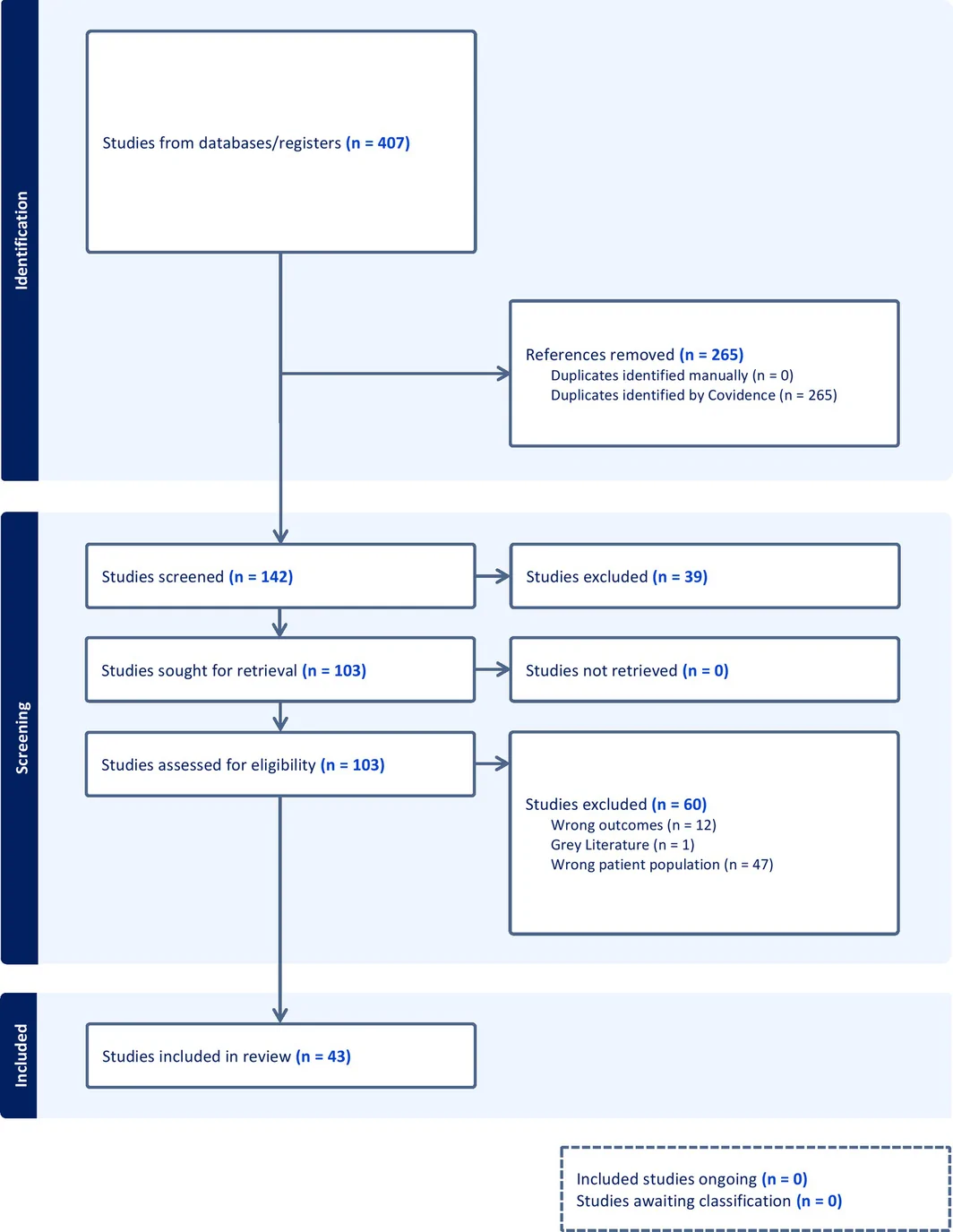
PRISMA flow.

Figure 1 outlines the PRISMA process followed during study screening and data extraction. Using Covidence, 265 duplicate articles were removed from the original 407 identified within the databases listed. A total of 142 article titles and abstracts were then screened. Of these, 39 were excluded, and 103 were read in full. Many of these articles discussed flexibility among those with psychotic disorders but were excluded because they either did not compare flexibility to a control group or compared the populations on variables other than flexibility. An additional 12 articles were excluded during the full text analysis for various reasons, including insufficient exploration of flexibility, discussing flexibility as a generic executive function variable, or conflating flexibility and inhibition. One paper from the gray literature was removed. A total of 43 studies remained once full text analysis was complete. Following this, a basic data extraction was conducted that assessed the articles' measures for flexibility, population, study type and outcomes. Articles were then grouped according to their flexibility type and assigned a subgroup according to the articles' theme.

Although the PRISMA search identified several relevant studies, many were only indirectly related to the central constructs of interest. This limited the extent to which the review could provide a definitive answer to the research question or support the development of an integrative model. This pattern likely reflects the novelty and lack of conceptual clarity of research linking flexibility to extreme overvalued beliefs and political violence more broadly. To ensure adequate coverage, citation mapping and consultation with subject matter experts were used to identify additional relevant literature. The review therefore combines systematic search procedures with narrative elements to accommodate the theoretical and methodological heterogeneity of the field. Despite this, however, the available studies were often still limited in quality or direct relevance to the core research aims. Given this scarcity, the results section draws on findings across multiple related domains to theorize potential connections between flexibility and extreme beliefs. The outcome of this approach is a largely descriptive account of how different forms of flexibility have been conceptualized and examined in relation to extreme beliefs or similar. Although this reduces replicability, transparency has been prioritized through detailed reporting of search and screening procedures wherever possible.

## 3 Results

### 3.1 Cognitive Flexibility

#### 3.1.1 Definitions

A small number of studies conceptualize cognitive flexibility as a metacognitive process associated with self‐reflection and moderation (Decety 2024; Osorio and Reyes 2023; Rollwage et al. 2018). However, most research adheres to a neurocognitive perspective, defining cognitive flexibility as an executive function that operates in conjunction with inhibition and working memory (Diamond 2013; Morris and Mansell 2018). Despite this distinction, both perspectives converge on the notion that cognitive flexibility involves dynamic processes of switching and updating during goal‐directed problem solving. Switching enables individuals to draw comparisons between similar task conditions, consider the effectiveness of alternatives, and reconfigure and restructure information. Similar to switching, updating refers to adapting, changing, or adjusting to new task demands and situational goals. Updating also involves the implementation of new strategies and updating mental representations (Buechner et al. 2021). These processes have been examined in relation to perspective‐taking, information processing, and adaptive problem‐solving strategies. Some definitions further describe the contexts in which cognitive flexibility can be applied, including goal‐directed behavior and cognition, problem‐solving, or when meeting the demands of a task or situation. Three additional widely cited, yet contentious (Cherry et al. 2021), criteria are outlined by Martin and colleagues (Martin and Anderson 1998; Martin et al. 1998; Martin and Rubin 1995). These include an awareness of what flexible responses are available; a willingness to be flexible; and confidence in executing a flexible response.

#### 3.1.2 Development and Brain Regions

Cognitive flexibility is one of the final major executive functions to develop in childhood, with early task switching abilities emerging from 2½ years of age (Diamond 2013). How cognitive flexibility develops and is applied over time remains less understood. While some research considers cognitive flexibility to be a mostly stable trait or disposition, others have suggested it to be context dependent and applied inconsistently across time and situation. As Cuevas and Dawson (2021) describe, it has traditionally been argued that the application of switching and updating is not specific to particular scenarios or conditions but is equally applied to the same degree across situations from the top‐down. In this sense, general cognitive control has been thought to override habitual, automatic behaviors or cognitive processes when prompted, regardless of context or scenario. More recently, cognitive flexibility has become more commonly viewed to be subject to change over time and triggered by environmental stimuli (Braem and Egner 2018; Cuevas and Dawson 2021). For example, positive intergroup interactions over time can reduce less ingroup‐centric worldviews and increase openness, creativity, and cognitive flexibility (Boin et al. 2020; Hodson et al. 2018).

The activation of various regions of the prefrontal cortex (PFC) has been linked to cognitive flexibility and executive functioning, particularly with relation to deliberation, cognitive control and subjective value processing (Tobeña 2021). Consequently, considerable research has explored the relationship between the PFC, cognitive flexibility and extreme beliefs. For example, reduced activation of the dorsolateral prefrontal cortex (dlPFC) has been associated with an increased willingness to fight and die for a belief and endorsement of violent confrontations (Decety 2024; Hamid et al. 2019; Tobeña 2021; Workman et al. 2020). Similarly, decision making involving sacred values is characterized by decreased dlPFC activation, as these beliefs function as moral imperatives that override conventional cost–benefit analysis (Hamid et al. 2019). Damage to the PFC is typically associated with impaired executive functioning, which, in turn, has been linked to an increased likelihood of reporting mystical experiences, such as encounters with the supernatural (Cristofori et al. 2016). Additionally, lesions in specific PFC regions have been implicated in the endorsement of extreme beliefs. For example, damage to the ventromedial prefrontal cortex (vmPFC) among Vietnam veterans has been associated with a greater acceptance of radical opinions, while lesions on the dlPFC are associated with greater acceptance of more radical opinions (Cuevas and Dawson 2021; Zhong et al. 2017). Collectively, these findings suggest a relationship between extreme beliefs, behaviors and cognitive inflexibility that may, at least in part, stem from disruptions in PFC functioning.

#### 3.1.3 Theoretical Mechanisms Linking Cognitive Flexibility to Extremism

Early theories of cognitive functioning in socio‐political contexts examined the relationship between uncertainty tolerance and cognitive rigidity, particularly among individuals with right‐wing political beliefs (Adorno 1950; Rokeach 1948, 1956; Sidanius 1985). Indeed, the *rigidity‐of‐the‐right* hypothesis, which posits that conservative individuals exhibit greater cognitive rigidity and lower tolerance of uncertainty, remains influential in contemporary research (for more, see Jost (2017) and van Prooijen and Krouwel (2017)). Studies have identified differences in executive functioning between liberals and conservatives, with liberals demonstrating greater updating, whereas conservatives have a greater ability to inhibit initial responses (Buechner et al. 2021, 2022). Other early evidence supported context theory, which suggested that political partisans tend to be more cognitively complex and actively engaged in politics, whereas moderates rely more on social conformity for political understanding, reflecting rigidity and close‐mindedness (Sidanius 1985).

This review found greater consensus for *extremism theory*, in that political partisans with the strongest beliefs engage with political events with cognitive inflexibility (Lyle and Grillo, 2020; Prichard and Christman 2020; Rollwage et al. 2018; van Prooijen and Krouwel 2017). Research has suggested that political partisans, regardless of political orientation, exhibit similar degrees of cognitive inflexibility (Zmigrod and Rentfrow 2020), and are also more likely to endorse violence to defend their beliefs (Zmigrod, Rentfrow, Zmigrod, and Robbins 2019). Indeed, those with extreme beliefs appear to operate with a high degree of certainty and self‐assurance, contrasting with flexible persons, whose critical and self‐reflective nature may restrict the willingness to justify extreme actions. Cognitive flexibility enables individuals to critically analyze multiple theories and sources of evidence; to reflect and update one's beliefs; and limits the use of one‐dimensional perspectives (Sambol et al. 2024). Conversely, a failure to engage in self‐reflection and updating can reinforce the perception that one's beliefs are absolute or objectively true, increasing the extremity in which moral decisions are made (Cuevas and Dawson 2021; Decety 2024; Osorio and Reyes 2023; Rollwage et al. 2018).

The proposed mechanisms linking cognitive flexibility to extreme beliefs remain largely theoretical. One key challenge in establishing empirical support is the distinction between “hot” and “cool” executive functions. Contemporary models of executive functioning differentiate “hot” executive functions, which operate in emotionally charged, context‐dependent situations, from “cool” executive functions, which are decontextualized and emotionally neutral (Zelazo 2015; Zelazo and Cunningham 2007). Since cognitive flexibility is classified as a “cool” executive function, its role in extreme beliefs, typically characterized by strong emotional elements (Rahman et al. 2020; Swann et al. 2024), becomes increasingly complex to define. Notably, many of the studies reviewed do not account for this distinction. As a result, the theoretical mechanisms proposed thus far should be interpreted with caution.

### 3.2 Psychological and Affective Flexibility

CF is generally applied to decontextualized tasks (e.g., chess problems) that elicit negligible amounts of emotive responses. When emotion is elicited, applying flexibility can activate unique regions of the PFC that are distinguishable from the areas activated in decontextualized tasks (Zelazo 2015). The orbitofrontal and ventromedial PFCs, for example, are associated with the processing of emotionally valenced content, whereas the dlPFC is associated with flexibility under cool, emotionally neutral conditions (Nejati et al. 2018; Salehinejad et al. 2021). It is therefore important to consider what has been referred to as affective flexibility when describing the mechanisms through which extreme beliefs develop.

#### 3.2.1 Flexibility Applied to Emotion

The literature revealed several applications of flexibility related to switching and updating in contextualized, affective conditions. Affective flexibility (AF), for example, is defined as a “specific type of cognitive flexibility that is used in the processing of affective material” (Genet et al. 2013). Applying AF enables individuals to better control emotional processes and problem‐solve effectively in emotionally charged situations. This, in turn, provides greater protection against rumination and improves the application of emotion regulation strategies (Genet et al. 2013). AF can also act as a protective factor against emotional manipulation, which is often used in radicalization processes (Haq et al. 2020). These techniques exploit emotional vulnerabilities, creating a sense of emotional dependency that reinforces radical beliefs and behaviors (Haq et al. 2020). By maintaining emotional balance, individuals are less likely to be swayed by emotionally charged propaganda (Howard et al. 2024; Naderer et al. 2024). Several related applications of flexibility related to AF also appear in the literature. Emotional flexibility and regulatory flexibility describe the ability to alternate between emotion regulation strategies to control emotions from the top‐down (Bonanno and Burton 2013; Mărcuș et al. 2024). Regulatory flexibility, however, emphasizes the ability to modulate or inhibit emotional responses based on situational demands. AF and the construct's derivations may indirectly contribute to wellbeing by enhancing the ability to adjust emotion regulation strategies according to situational demands, rather than serving as a regulation strategy in itself.

Psychological flexibility (PF) was also identified as an application of flexibility within contextualized scenarios. PF is characterized by mindfulness and acceptance of all internal thoughts and feelings that enables goal‐directed behavior (Cherry et al. 2021; Constantinou et al. 2021; Oliver et al. 2012). The higher order ability involves the ability to tolerate and accept negative thoughts and emotions, as well as behaving in accordance with personal values and long‐term goals. Similar to CF, PF engages switching and updating in various capacities, enabling individuals to adapt to emotionally valenced scenarios. Conversely, psychological inflexibility often manifests through the overuse of a singular coping mechanism such as experiential avoidance, in which individuals actively disengage from certain aspects of their internal experience (Constantinou et al. 2021; Kashdan et al. 2020). Over time, avoidance strategies (e.g., distraction, disengagement or substance use) (Dawson and Golijani‐Moghaddam 2020) may generalize across contexts through repeated use, reinforcing rigid cognition and behavior and reducing adaptive coping in response to distress (Kashdan and Rottenberg 2010). This is not to say that experiential avoidance is the antithesis to PF, as PF entails the activation of a range of coping mechanisms, including experiential avoidance (Dawson and Golijani‐Moghaddam 2020). Another parallel to CF is the conflicting evidence regarding the stable vs. dynamic nature of PF (Constantinou et al. 2021). Findings from this review support the notion that PF can be increased over time and is context dependent.

#### 3.2.2 Application to Extreme Beliefs

Research is limited on the role of AF and PF in the formation and maintenance of extreme beliefs. However, it is evident that those who are capable of recognizing which cognitive abilities to use for a particular emotionally valenced situation are better protected against radicalization. The inability to manage emotional responses to threatening or distressful social events is associated with psychological distress, intolerance of uncertainty and need for closure. Such factors can motivate them to identify with groups that provide structure and clarity (Deverson et al. 2025; Hogg et al. 2010, 2007; Kashdan and Rottenberg 2010; Schulz et al. 2020). Individuals with pre‐existing insecure or fragmented identities may experience general emotional instability, making them more susceptible to group ideologues (Ozer et al. 2023; Pilkington 2023). Group identification like this can lead to the adoption of restrictive, authoritarian beliefs, or a willingness to use violence in defence (Hogg and Adelman 2013; Swann et al. 2024). Additionally, maladjustment to threat and uncertainty can increase emotional reactivity and impulsivity, which can increase the likelihood of engaging in risky group‐based behaviour (Elzesser et al. 2023). This was evident during the COVID‐19 pandemic, whereby those with greater PF had more positive psychosocial and wellbeing outcomes under the cognitive strain of lockdowns and limited freedom (Dawson and Golijani‐Moghaddam 2020). PF also mediated the relationship between conspiracy theory beliefs and the likelihood of adhering to government regulations during the pandemic (Acar‐Burkay and Cristian 2022; Constantinou et al. 2021).

Given the intense emotional connection people share with rigidly held group beliefs (Rahman et al. 2020), it is likely that a high degree of AF is required to problem solve effectively. Rigid overreliance on emotional, intuitive reasoning abilities when experiencing threatening or ego‐boosting stimuli may increase the risk of developing extreme beliefs, as they can increase ignorance to heuristics and bias (Pytlik et al. 2020). These people may be more difficult to convince that their beliefs are irrational or illogical as they perceive reality through black‐and‐white filters. Empathy and consideration of others' perspectives, a component of affective flexibility, may be limited here. While empathy can foster understanding and reduce conflict, its absence can lead to dehumanization and support for violent extremism (Howard et al. 2024). AF, therefore, may promote understanding and reduce the dehumanization of outgroups. This suggests that the ability to process, accept or disengage from the emotion of a situation, and engage in effortful, cognitive processes to problem‐solve is necessary to reduce the likelihood of developing extreme beliefs.

### 3.3 Belief Flexibility

#### 3.3.1 Definitions

A third major application of flexibility found in the delusion literature is belief flexibility (BF), which refers to the ability to reflect on one's own beliefs, change them in light of reflection and evidence, and generate and consider alternative theories (Colbert et al. 2010; Eisenacher and Zink 2017; Sulik et al. 2023). Although extreme beliefs are characteristically non‐delusional (Rahman et al. 2020), belief inflexibility is present among some within the non‐clinical population (Eisenacher and Zink 2017; Prochwicz et al. 2018). It is possible, therefore, that this application of inflexibility can uniquely contribute to extreme belief formation and, perhaps more significantly, their maintenance. Similar to the process of updating in relation to CF, key to belief flexibility is the concept of evidence integration. As the world changes or presents novel evidence, the flexible individual can critically assess this new evidence in light of their beliefs and, if appropriate, update their beliefs through integration. Importantly, belief flexibility involves the ability to integrate new disconfirmatory evidence, as well as confirmatory evidence. As in the case of conspiracy theory beliefs, being too open to evidence that consistently confirms a narrative may contribute to the conviction in which these beliefs are perpetuated. Greater belief flexibility must therefore involve a healthy balance between openness to experience, critical analysis, and evidence integration.

Belief inflexibility or rigidity is characterized by a resistance to incorporating new or contradictory evidence, which can manifest as a bias against disconfirmatory evidence (BADE) (Bronstein and Cannon 2017; Ward and Garety 2019). Individuals with rigid beliefs may struggle to revise their perspectives even when confronted with compelling counterevidence, instead reinforcing their own fixed and inflexible perspectives. In this sense, belief flexibility encourages switching and updating or “playing” with ideas, thoughts and evidence, rather than specific cognitive abilities or strategies. Switching and updating in this manner enables the individual's beliefs to more accurately reflect themselves and the world around them, which in itself may be considered a problem‐solving strategy. The more a belief is revised when presented with confirmatory or disconfirmatory evidence, the greater the possibility they can represent the nuanced and complex reality of sociopolitical phenomena.

Underlying current delusion research paradigms is the argument that delusions and delusion‐like beliefs in non‐clinical populations are a result of cognitive biases like the BADE and jumping to conclusions. This same theme can be observed throughout the radicalization and extreme belief literature identified in this review. This relationship has been called into question, however. In Sulik et al. (2023), when accounting for careless responses, the relationship strength between measures of cognitive biases (e.g., BADE and Beads Task) and delusion‐like beliefs became significantly weaker. Other research has not been able establish a relationship between belief flexibility and delusional beliefs among high‐delusion‐prone participants (Howe et al. 2018). Given the similarities in rigidity between delusional and extreme ideological beliefs, this evidence could suggest that the assumed link between cognitive biases, rigidity and extreme beliefs may be overstated or context dependent.

#### 3.3.2 Application

Despite the similarities between CF and BF, the BF literature offers unique insights into group dynamics and flexibility during belief formation and maintenance. An inability to integrate evidence is associated with less cognitive insight into the validity of a belief; radical political attitudes; racial prejudice and dogmatism; and diminished accuracy in predicting another's moral character (Bronstein et al. 2017; Rollwage et al. 2018; Zhao and Cannon 2024). Furthermore, some beliefs prove more rigid and inflexible than others. This is particularly apparent when beliefs contain a significant affective component which demonstrates the role of emotion in belief formation (Cesur et al. 2023; Colbert et al. 2010; Zhu et al. 2021). Theoretically, evidence integration difficulties may exist when the belief relates to the individual's identity (i.e., ego or self‐esteem); relieves distress and provides certainty (i.e., existential dread, an afterlife, etc.); or is used to explain complex social phenomena (i.e., political unrest, pandemics, etc.).

Belief inflexibility may be characteristic of groups that demonstrate *groupthink*, or the tendency of group members to conform to a consensus viewpoint, often at the expense of critical thinking and individual creativity. Groupthink often leads to suboptimal decision‐making as it suppresses dissenting opinions and critical evaluation (Juni and Eckstein 2015; Priyanto and Wening 2024). Groups that can abandon rigid perspectives and rules in favor of more dynamic approaches tend to achieve higher decision accuracies (Juni and Eckstein 2015). Individual abstract reasoning abilities under conditions of group conformity may also be restricted here. Low BADE and belief flexibility have been associated with greater scientific reasoning skills and open‐mindedness; and negatively correlated with CT beliefs and fast, intuitive reasoning (Erceg et al. 2022; Georgiou et al. 2022). Additionally, those with greater fundamentalist religious beliefs are less likely to integrate evidence and update their religious beliefs (Cuevas and Dawson 2021).

## 4 Discussion

### 4.1 Summary and Overview

Research consistently indicates that political partisans exhibit greater cognitive rigidity than moderates (van Prooijen and Krouwel 2017; Zmigrod and Rentfrow 2020). Additionally, different forms of flexibility (i.e., cognitive, affective, psychological and belief) have been proposed as protective factors that reduces susceptibility to radicalization and violent extremism. However, the theoretical mechanisms underlying these constructs remain insufficiently understood. This review therefore examined the diverse conceptualizations and applications of *flexibility*, which broadly refers to the ability to adapt thoughts, emotions and cognitive strategies to enable goal‐directed problem solving (Cherry et al. 2021; Diamond 2013; Martin and Rubin 1995). Despite limited empirical evidence on the subject, the findings emphasize the relevance of cognitive, affective, psychological and belief flexibility in shaping beliefs relating to authoritarianism, prejudice, and the endorsement of violence. An underlying feature of all these flexibilities frequently reported across the various literatures was the capacity to *update and switch* between thoughts, emotions, strategies and perspectives. Accordingly, updating and switching can be applied in various ways during belief formation and maintenance. Broadly, an individual may demonstrate flexibility through critical assessment and integration of new evidence into a belief where necessary, while considering a situation's contextual features. Flexibility may also be applied while being tolerant and accepting of uncertainty, difference and negative affect arising from alternative beliefs, evidence or ideas. It should be noted that these are but a few examples of how flexibility can be applied.

### 4.2 Core Features of Cognitive and Affective Flexibility

An important finding was the distinction between the two “hot” and “cool” categories that reflect distinct neural processes and uniquely protect against the formation and maintenance of extreme beliefs (Salehinejad et al. 2021; Tobeña 2021; Zmigrod, Rentfrow, and Robbins 2019). The first, “cool” flexibility, is related to the strategies the individual uses to problem‐solve under emotionally neutral, decontextualized conditions, and is associated with cognitive flexibility (CF). The second category relates to “hot” flexibility, or flexibility in the context of emotionally valenced situations, and aligns more closely with affective flexibility (AF). Where CF can be applied to problem‐solve in the context of emotionally neutral beliefs (e.g., the sun will rise tomorrow), it is likely that AF is more relevant to beliefs that are personally meaningful and that contain significant emotional elements (Cesur et al. 2023; Colbert et al. 2010; Genet and Siemer 2011; Zhu et al. 2021) Figure 2 illustrates this difference.

**FIGURE 2:**
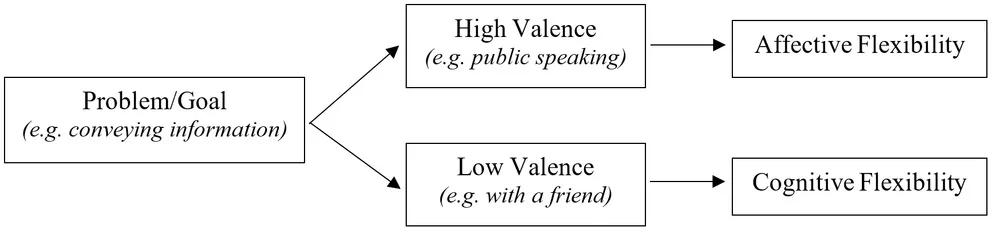
Difference in application of affective and cognitive flexibility.

In line with the findings of this review, we propose that flexibility operates in a dual capacity. Specifically, CF is expected to be more strongly associated with belief development under neutral conditions, whereas AF is likely to play a greater role in emotionally valenced contexts. Accordingly, cognitive and affective flexibilities may be considered as two core variants of flexibility, involving switching and updating abilities that are differentially activated in the prefrontal cortex depending on the emotional characteristics of the context.

Other flexibility constructs discussed in this review, including psychological, belief, emotional, and regulatory flexibility, may be understood as applied or domain specific expressions of these core processes. In this framework, such constructs apply switching and updating toward particular problems. For example, PF directs these processes toward alternative self‐perspectives in order to promote tolerance and acceptance, particularly in the context of emotion regulation in mental health. When attributing causality to complex social phenomena, belief flexibility appears to encourage switching and updating toward evidence integration processes. Although all forms of flexibility here appear to contribute to belief development in varying capacities, we suggest that research on extreme belief formation and maintenance should prioritize cognitive and affective flexibility. These constructs most directly capture the underlying switching and updating processes that define flexibility.

### 4.3 Future Research

This distinction between CF and AF may help explain why those with extreme beliefs can exhibit cognitive flexibility in some domains yet demonstrate rigidity when confronted with emotionally charged situations, such as when their beliefs are challenged or criticized, or when they perceive uncertainty, threat, or injustice. However, this explanation only adds further complexity to the role of cool, decontextualized CF in extreme belief formation or maintenance. Research directly relating CF with a willingness to fight/die for a belief (Schumann et al. 2022; Zmigrod 2022; Zmigrod, Rentfrow, Zmigrod, and Robbins 2019) does not account for this distinction. Future studies could explore how these distinct forms of flexibility function at different stages of belief development. For example, the early stages of belief formation may involve more CF, during which period the belief has yet to develop significant emotive elements. Contrastingly, belief maintenance may depend more on AF, where the individual has invested more emotional resources into the belief. Regardless, a more nuanced understanding of these processes is necessary to clarify how flexibility influences ideological extremism.

Additionally, most flexibility studies identified in this review focused on flexibility's relationship with authoritarianism, conspiracy theory beliefs, ethnocentrism, and prejudice. However, these constructs are not necessarily interchangeable with extreme beliefs, which specifically involve the endorsement or use of violence (Buechner et al. 2021). While violent extremism has previously been considered to be the natural evolution from authoritarian opinions (e.g., the Staircase Model) (Moghaddam 2005), this relationship is complex and not necessarily linear (Pilkington 2023). The two‐pyramids model, for example, describes differences in radicalization of opinion and radicalization of action (McCauley and Moskalenko 2017). Evidence for the relationship between cognitive flexibility and extreme beliefs is therefore limited to a handful of studies that include measures of extreme beliefs that endorse the use of violence. Future research would therefore benefit from applying various measures of flexibility to extreme beliefs.

### 4.4 Limitations

Several limitations of this review should be acknowledged. Initially, an iterative search strategy following PRISMA guidelines was intended to be used to increase transparency and replicability. Following several attempts, it became apparent that any search strategy was too broad to cover the relevant information on flexibility, or too narrow to cover flexibility applied to extreme beliefs. As a result, a manual search strategy was used in addition to the results generated through PRISMA, and a hybrid narrative scoping review format was deemed the most appropriate approach for this topic. Unlike systematic or scoping reviews, narrative reviews are more susceptible to author bias and have weaker methodological transparency. Consequently, the findings presented in this review should be interpreted with care, as relevant studies may have been unintentionally overlooked or excluded. Additionally, this review did not aim to provide a comprehensive account of all applications of flexibility. Instead, it focuses on the most pertinent and empirically supported applications identified in the literature. Future research may benefit from a systematic approach to further refine and expand on these findings.

### 4.5 Conclusion

This review highlights the significant role of flexibility in understanding and addressing extreme beliefs. The capacity to engage in flexible thinking by keeping oneself open to various problem‐solving strategies mitigates the cognitive rigidity associated with extreme beliefs, including a willingness to fight and/or die in defense of such beliefs. The findings suggest that flexibility operates through both hot affective and cool neutral cognitive functions, the activation of which depends on the degree of emotion involved. These abilities are not associated with one particular problem‐solving strategy but rather facilitate a range of adaptive responses, including evidence analysis and integration; critical thinking; emotion regulation; and tolerance of uncertainty. Additionally, the review further highlights gaps in the existing theoretical mechanisms linking flexibility to extremism thus far, particularly the need to distinguish cool cognitive and hot affective flexibilities. Future research should further investigate the dynamic interplay between cognitive and affective flexibility to clarify the mechanisms underlying the rigidity characteristic of extreme belief formation and maintenance.

## Author Contributions

**Sebastian Deverson:** conceptualization, data curation, methodology, project administration, writing – original draft, writing – review and editing. **Paul Delfabbro:** conceptualization, methodology, project administration, supervision, validation, writing – review and editing. **Ryan Balzan:** conceptualization, methodology, supervision, validation, writing – review and editing.

## Funding

The authors have nothing to report.

## Conflicts of Interest

The authors declare no conflicts of interest.

## Data Availability

Data sharing not applicable to this article as no datasets were generated or analyzed during the current study.
